# Gelatin-based perfusable, endothelial carotid artery model for the study of atherosclerosis

**DOI:** 10.1186/s12938-019-0706-6

**Published:** 2019-08-07

**Authors:** Ruomeng Chen, Bo Wang, Yaxiong Liu, Jiankang He, Rong Lin, Dichen Li

**Affiliations:** 1 State Key Laboratory for Manufacturing Systems Engineering, Xi’an Jiaotong University, Xi’an, China; 2 Departments of Pharmacology, School of Basic Medical Sciences, Xi’an Jiaotong University Health Science Center, Xi’an, China

**Keywords:** Carotid artery model, Wall shear stress, Carotid geometry, Endothelial cell, Gelatin, Atherosclerosis

## Abstract

**Background:**

Carotid artery geometry is important for recapitulating a pathophysiological microenvironment to study wall shear stress (WSS)-induced endothelial dysfunction in atherosclerosis. Endothelial cells (ECs) cultured with hydrogel have been shown to exhibit in vivo-like behaviours. However, to date, studies using hydrogel culture have not fully recapitulated the 3D geometry and blood flow patterns of real-life healthy or diseased carotid arteries. In this study, we developed a gelatin-patterned, endothelialized carotid artery model to study the endothelium response to WSS.

**Results:**

Two representative regions were selected based on the computational fluid dynamics on the TF-shaped carotid artery: Region ECA (external carotid artery) and Region CS (carotid sinus). Progressive elongation and alignment of the ECs in the flow direction were observed in Region ECA after 8, 16 and 24 h. However, the F-actin cytoskeleton remained disorganized in Region CS after 24 h. Further investigation revealed that expression of vascular cell adhesion molecule-1 (VCAM-1) and intercellular adhesion molecule-1 (ICAM-1) was greatly increased in Region CS relative to that in Region ECA. The physiological WSS in the carotid artery system was found to stimulate nitric oxide (NO) and prostacyclin (PGI_2_) release and inhibit endothelin-1 (ET-1) release after 24-h perfusion experiments. The effective permeability (E.P) of fluorescein isothiocyanate (FITC)–dextran 40 kDa in Regions ECA and CS was monitored, and it was found that the turbulence WSS value (in Region CS) was less than 0.4 Pa, and there was a significant increase in the E.P relative to that in Region ECA, in which laminar WSS value was 1.56 Pa. The tight junction protein (ZO-1) production was shown that the low WSS in Region CS induced ZO-1-level downregulation compared with that in Region ECA.

**Conclusions:**

The results suggested that the gelatin-based perfusable, endothelial carotid artery model can be effective for studying the pathogenesis of atherosclerosis by which flow dynamics control the endothelium layer function in vitro.

**Electronic supplementary material:**

The online version of this article (10.1186/s12938-019-0706-6) contains supplementary material, which is available to authorized users.

## Background

Carotid artery diseases such as atherosclerosis are characterized by artery wall fibrosis and lipid accumulation,which involve the endothelial cell (EC) dysfunction [1]. EC dysfunction is mediated by haemodynamic forces that are dependent on the carotid arterial geometry [2, 3]. For example, carotid stenosis is prone to occur in regions of curvature and bifurcation [4]. Therefore, a comprehensive understanding of the carotid artery pathophysiology necessitates the study of endothelial dysfunction based on the carotid artery geometry.

Due to the difficulty in studying EC dysfunction in vivo, three-dimensional (3D) tissue-engineered vascular models were investigated. These models can provide a multifactorial environment that is critical for EC functioning, but they often fail to reproduce carotid artery features, including geometry and blood perfusion, which can affect endothelial functions [5]. As EC dysfunction and flow disturbance at the early stage can manifest into atherosclerosis complications, it is important to develop a perfusion-based carotid artery model with carotid artery geometry to better understand pathophysiology [2].

Microfluidics models are among the earliest in vitro models that have advanced our understanding of intercellular interactions under fluid flow [6]. Most microfluidics systems have been fabricated with polydimethylsiloxane (PDMS)-based photolithographic techniques. However, PDMS cannot provide a physiologically appropriate environment with spatial heterogeneity similar to that found in the native vessel [7]. Furthermore, microfluidic devices are faulty due to non-physiological flows resulting from the rectangular cross sections of the microchannels [8]. ECs cultured with hydrogel have been shown to exhibit like behaviours similar to those in vivo [9, 10]. It is therefore necessary to explore a hydrogel model with complete carotid artery geometric characteristics that can meet the requirements for a carotid artery model in terms of material biological properties and complex structural characteristics. Three-dimensional (3D) bioprinting techniques have been developed using an in vitro tissue model with hydrogels [11]. However, most 3D bioprinting techniques cannot replicate the carotid artery geometry (e.g. the bifurcation, curvature and different diameters) or flow perfusion. Two main strategies are available for forming perfusable microchannels using 3D bioprinting techniques. One strategy involves the use of a sacrificial material. Miller et al. generated a vascular network with printed sacrificial bioglass, cast it into a hydrogel and then dissolved the bioglass in water [12]. The other strategy involves tube-like structures. Zhang et al. fabricated a vessel-like microfluidic channel using a co-axial nozzle system that enabled the direct printing of a hollow vascular channel in a 3D hydrogel [13]. However, these techniques could not generate a geometry that is sufficiently similar to that of the carotid artery. Self-assembly is the most promising technique for building a tissue-engineered carotid artery. Norotte et al. employed agarose rods, which are tubular structures with controllable tube diameters, as templates and found that wall thickness and branching patterns could be replicated by the deposition and fusion of multicellular spheroids. Unfortunately, fluid perfusion has not been applied to verify that the bifurcated structure manufactured using this process has suitable mechanical properties to realize the dynamic culturing of ECs under physiological flow conditions without leakage (Additional file 1).

A manufacturing method that we have studied provides a flexible approach to fabricate a branched microfluidic network with circular cross sections in a hydrogel by combining micromoulding and enzyme-induced crosslinking [14]. With this technology, an engineered carotid artery with qualified mechanical properties and a complete geometry can be manufactured, and a perfusion system can be assembled to construct an in vitro carotid artery system. Here, we used the in vitro carotid artery system to study the effects of hydrodynamic behaviours on ECs. In particular, we aimed to understand the aetiology of atherosclerosis by evaluating the mechanisms underlying the effects of WSS on EC morphology, endothelial permeability, protein secretion and endothelial vasoactive substance release in the carotid artery system.

## Methods

### Perfusable, endothelial carotid artery model preparation

**Fig. 1:**
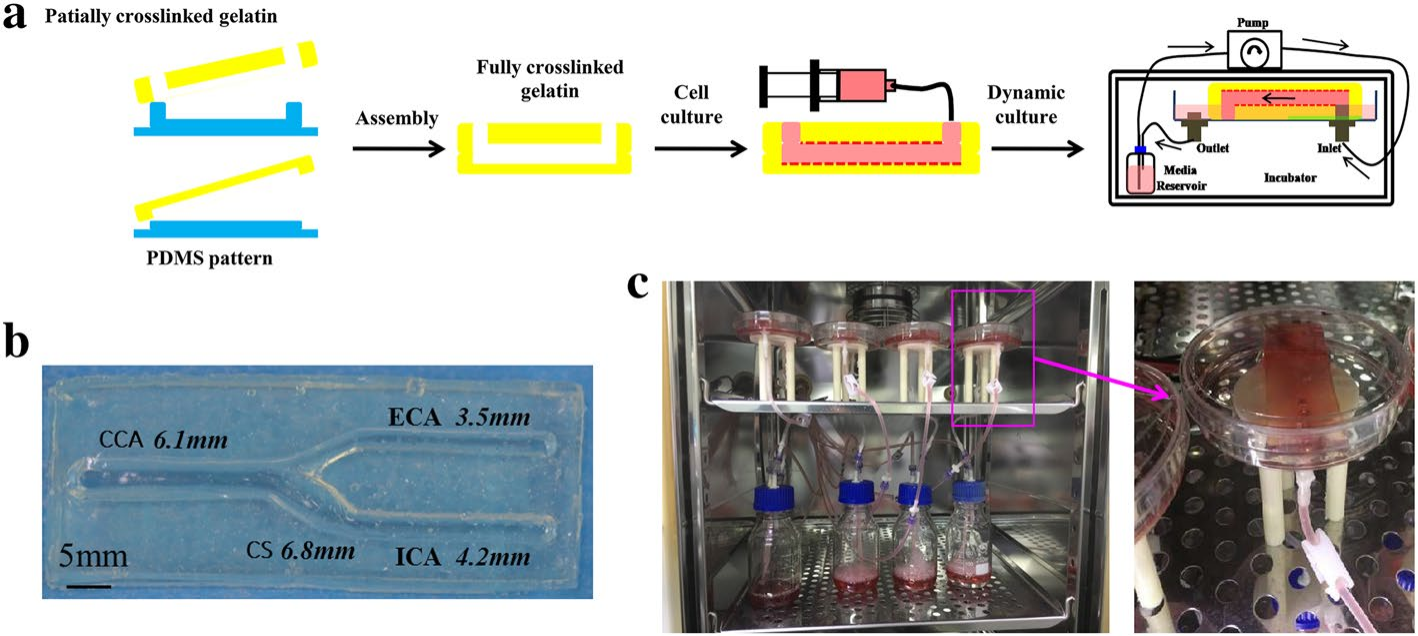
Preparation of perfusable, endothelial carotid artery model. **a** Overview of the carotid artery system fabrication process. **b** Gelatin-based carotid artery model. **c** Actually assembled carotid artery system

A tuning fork-shaped (TF-shaped) gelatin-based carotid artery model was developed in this work [15]. The diameters of the TF-shaped carotid artery were 6.1 mm, 4.2 mm, 3.5 mm and 6.8 mm for the common carotid artery (CCA), internal carotid artery (ICA), external carotid artery (ECA) and carotid sinus (CS), respectively. The manufacturing process for the carotid artery models is shown in Fig. 1a. Briefly, stereolithography (SL) was used to fabricate a resin mould with semicircular microchannels, which were transferred to a PDMS mould. The mTG/gelatin solution was piped into the PDMS mould to induce gelation to form a monolayer microfluidic hydrogel. Two partially crosslinked hydrogel layers with semicircular channels were assembled and fully crosslinked to form a closed circular 3D carotid artery model, as shown in Fig. 1b. ECs (ATCC-1730, Manassas, USA, 2.0 × 10^5^ cells/ml) were uniformly spread on the internal surface of the carotid artery model by rotating implantation. Then, the carotid artery model was statically cultured in DMEM/low glucose medium (Hyclone, USA) supplemented with 10% fetal bovine serum (FBS, Gibco, USA) and 100 U/ml penicillin–streptomycin (Gibco, USA) in a humidified atmosphere with 5% CO_2_ at 37 °C until the 5th day. The ECs were evenly distributed inside the model channel, and the function and activity of the ECs were optimal (SF 1). For the flow experiments, the models were connected to a perfusion loop composed of individual media reservoirs and a peristaltic pump (Harvard Apparatus, USA), as shown in Fig. 1c and Additional file 2: Video S1. The reservoir contained ECs culture media containing 10% fetal bovine serum and 1% penicillin streptomycin.

### Finite element simulation

The COMSOL5.3 Multiphysics CFD module was used to predict the fluid flow profile, streamlines and WSS distribution inside the carotid artery geometry. The laminar flow module was used, which assumes incompressible flow and no turbulence but does not neglect the inertial term in the Navier–Stokes law. The viscosity of the fluid (culture media) is 1.13 cP, and the density is 1000 kg/m^3^ at 37 °C [16]. A flow rate of 5.6 ml/s was set on the inlet for the physiological velocity [17]. An entrance length of 1 m was chosen to provide a fully developed flow at the entry of the geometry, and the outlet pressure was set to zero. On the outer boundary of the geometry, excluding the inlet and outlets, a no-slip boundary condition was imposed.

### Rearrangement of the F-actin cytoskeleton

After the fluid dynamic treatment, a fluorescence microscope (Ti-S, Nikon, Japan) was used to visualize the F-actin cytoskeleton. The confluent cell monolayers in the models were fixed with 3.7% paraformaldehyde (Sigma-Aldrich, USA) for 10 min and permeabilized with 0.1% Triton X-100 (Sigma-Aldrich, USA) for 20 min. The cells were washed with PBS between each of the steps and were finally stained with 1% Actin-Tracker Green (Invitrogen, USA) solution in 1% BSA and 0.1% Triton X-100 for processing by immunofluorescence microscopy.

### Activation of endothelial vasoactive substances

The medium in the carotid artery system was collected at 8, 16 and 24 h and stored at − 80 °C until the endothelial vasoactive substances were measured. The nitric oxide (NO) production in the culture medium was measured by the Griess method, using an NO assay kit (Beyotime Biotech Inc., Jiangsu, China). NO concentration was determined by measuring the absorbance at 540 nm on a microplate reader (Thermo, USA). The nitrite concentration was calculated with reference to a standard curve of sodium nitrite generated from known concentrations. The amount of prostacyclin (prostaglandin PGI_2_) produced, calculated as the concentration of the stable hydrolysis product 6-keto-prostaglandin (6 k-PG) F1α, was determined in duplicate using a Prostacyclin EIA kit (Enzo Life Sciences, Farmingdale, NY, USA) [18]. The endothelin-1 (ET-1) concentrations were determined in duplicate by the Endothelin-1 EIA kit (Enzo Life Sciences, Farmingdale, NY, USA) following the manufacturer’s protocol [19]. The results were normalized to cell number in all experiments.

### Quantitative real-time PCR analysis

Gene expression was analysed by real-time polymerase chain reaction (PCR). To analyse the expression of vascular cell adhesion molecule-1 (VCAM-1) and intercellular adhesion molecule-1 (ICAM-1), total RNA from the cells in Regions ECA and CS was extracted by Trizol isolation, and cDNA was synthesized using the TaqMan reverse transcription (RT) Master Mix kit according to the manufacturer’s protocol. Real-time PCR analysis was performed with an Opticon Real-Time PCR Machine (Bio-Rad, CA, USA) using the SYBR Green PCR Master Mix Reagent Kit (Takara Biotechnology, Dalian, China). Each reaction mixture (total volume 20 μL) contained cDNA (equivalent to 100 ng RNA), 200 nM deoxyribonucleotides, each primer at 800 nM and 0.5 U GoTaq polymerase (Biotools B&M Labs, Madrid, Spain). The cycling conditions were as follows: incubation for 3 min at 95 °C followed by 35 cycles of 95 °C for 30 s, 55 °C for 45 s and 72 °C for 45 s.

The relative quantitation of the gene expression was determined by the ^ΔΔ^CT method, and GAPDH was used as a control. For these analyses, we used the specific primers shown in ST 1.

### Measurement of permeability

The permeability coefficient of the carotid artery system was quantified by introducing 40 kDa dextran conjugated with fluorescein isothiocyanate (FITC, Sigma-Aldrich, USA) into the endothelial carotid artery derived from the perfusion experiments to capture time-sequential images of FITC–dextran diffusion through the endothelial barrier. After the aspiration of the cell culture medium, the FITC–dextran solution was added (~ 2 ml) at the inlet of the endothelial carotid artery to perfuse the intraluminal space. Fluorescence images were obtained every minute with an inverted fluorescence microscope (Ti-S, Nikon, Japan) using a 1× objective. We calculated the effective permeability (E.P) by using Fick’s law, as described elsewhere [20, 21]. The permeability coefficient can be calculated using the equation below, assuming that the cross-sectional shape of the blood vessels is circular:

$$ E \cdot P = \frac{W}{T} \times \frac{{I_{1} - I_{0} }}{{I_{0} - I{}_{b}}} $$

where $I_{b}$ is the background intensity at the start, $I_{0}$ is the mean fluorescence intensity of a specific portion at the start, $I_{1}$ is the mean fluorescence intensity at the same portion after 10 min, $W$ is the width of the channel in centimetres and $T$ is the total time in seconds. During the fluorescence imaging, the fluorescence intensity in the intravascular region ($I_{0}$) was held constant. The fluorescent images were analysed with ImageJ software (National Institutes of Health).

### Statistical analysis

All values are expressed as the mean  ±  standard error of the mean (SEM) and were analysed using SPSS software (SPSS for Windows version 16.0, USA). All experiments were repeated at least three times. Differences between mean values of normally distributed data were evaluated by one-way ANOVA or two-way ANOVA followed by Tukey’s post hoc test. *P* <  0.05 was considered to indicate a statistically significant difference between groups.

## Results

### Computational fluid dynamics in the TF-shaped carotid artery

**Fig. 2:**
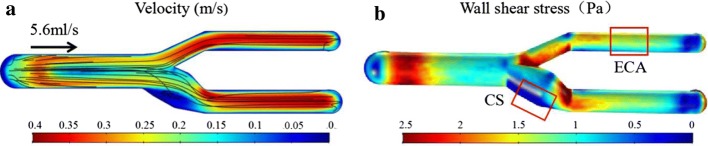
Computational fluid dynamics on the TF-shaped carotid artery. **a** Flow velocity simulation. **b** Wall shear stress simulation

The velocity and WSS profiles of the TF-shaped carotid artery model are presented in Fig. 2. The laminar flows in the CCA transform to turbulent flow at the CS due to the bifurcation, curvature and taper. Then, the streamlines at the ICA and ECA revert to laminar flow. As a result, the lowest velocity was found in the CS, and the streamlines in the CS showed a distinct eddy (Fig. 2a). Moreover, the turbulent flow in the CS created a lower WSS in the whole carotid artery model (Fig. 2b). Therefore, two representative regions of the model were selected for subsequent studies: Regions ECA and CS. The characteristics of Region ECA were laminar flow and WSS greater than 1 Pa (1.56 ± 0.013 Pa), and those of Region CS were turbulence and WSS less than 1 Pa (0.42 ± 0.027 Pa).

### EC morphologic response to carotid artery haemodynamics

**Fig. 3:**
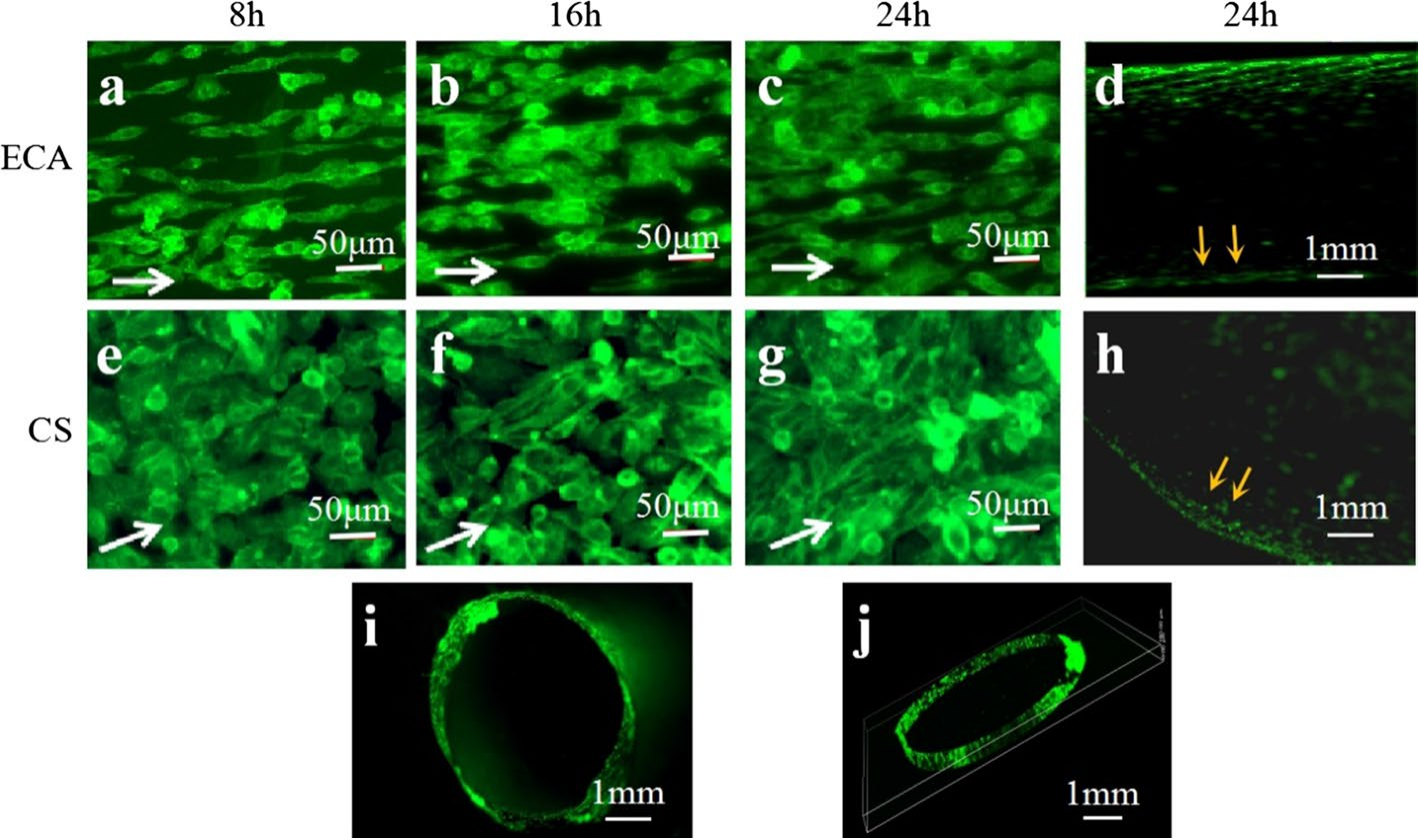
ECs morphology in the carotid artery model. ECs morphology in Region ECA after dynamic experiment 8 h (**a**), 16 h (**b**) and 24 h (**c**). **d** Macroscopic distribution of ECs in Region ECA. ECs morphology in Region CS after dynamic experiment 8 h (**e**), 16 h (**f**) and 24 h (**g**). **h** Macroscopic distribution of ECs in Region CS. **i**, **j** Distribution of ECs in cross section of Region ECA. (The white arrows point in the direction of flow.)

In Region ECA, progressive cell alignment in the laminar flow direction was observed at 8 h, which was followed by further cell elongation, alignment and proliferation at 16 and 24 h (Fig. 3a–c). ECs exposed to turbulent WSS for 8 h were significantly rounder, as shown in Region CS (Fig. 3e). When the ECs were exposed to turbulent WSS for 16 h, disorganization of the F-actin cytoskeleton was observed, as shown in Fig. 3f. Although elongation in the flow direction was observed for a few cells in Region CS at 24 h, the F-actin cytoskeleton remained disorganized under turbulent flow (Fig. 3g). Most importantly, the round ECs were found in Region CS. The ECs shape index (SI) was quantified as shown in SF 2. The morphological differences in ECs in the carotid artery model are caused by a combination of fluid state, wall shear stress and time.

After 24 h of dynamic perfusion, the ECs were distributed evenly on the ECA channel wall (Fig. 3d). However, the ECs distributed on the wall of Region CS were not uniform and showed cell aggregation, as highlighted by the yellow arrows in Fig. 3h. The ECs in Region ECA fully and evenly covered the surface, and transverse sectioning revealed that they formed an endothelialized monolayer, as shown in Fig. 3i, j.

### EC functional response to carotid artery haemodynamics

**Fig. 4:**
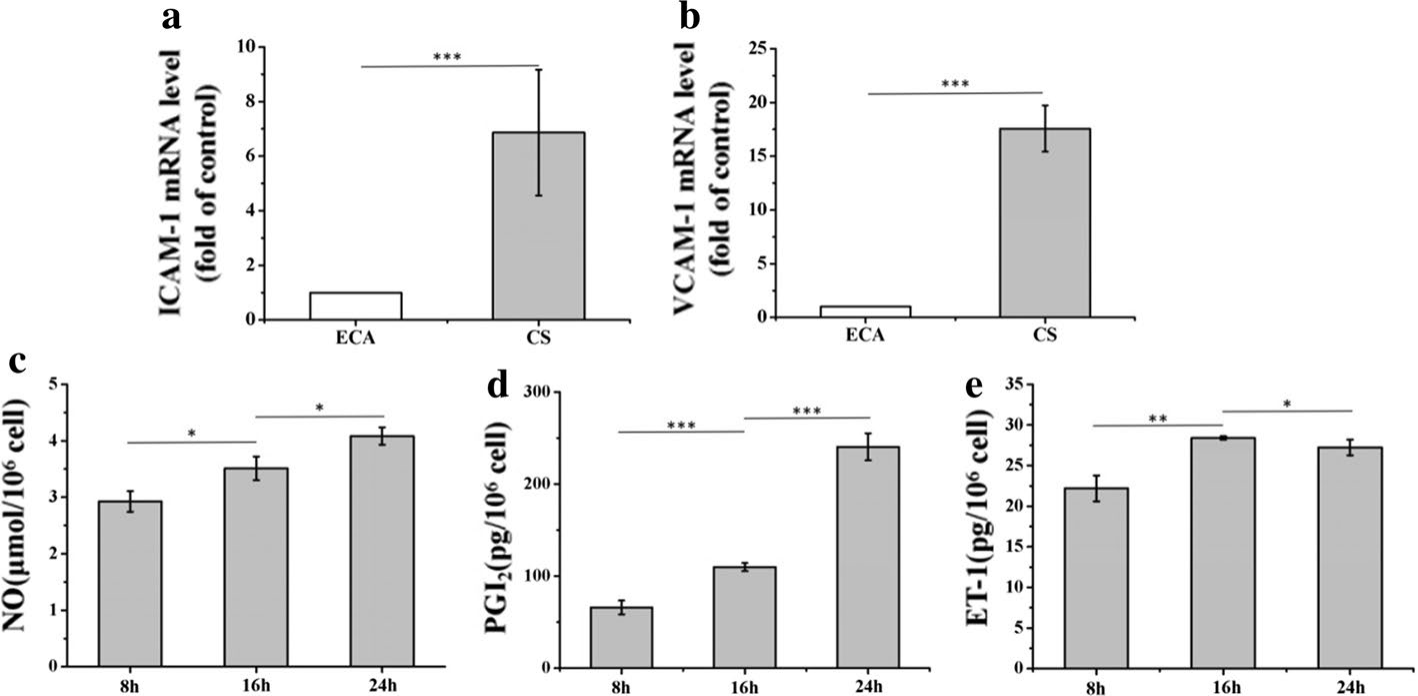
ECs function’ response to carotid artery hemodynamics. The expression of ICAM-1 (**a**) and VCAM-1 (**b**) was studied in the Regions ECA and CS after 24-h perfusion experiment. The vasoactive substances NO (**c**), PGI_2_ (**d**) and ET-1 (**e**) released after 8, 16 and 24 h. (Data presented are the mean ± SEM, *n* = 4, *P < 0.05, **P < 0.01, ***P < 0.001.)

The expression of ICAM-1 and VCAM-1 in Regions ECA and CS was studied in carotid artery models after 24 h of the perfusion experiment. As shown in Fig. 4a, b, the ECs in Region CS had greater expression of ICAM-1 and VCAM-1 than did those of Region ECA, which demonstrated that the WSS generated by the blood flow exerted direct mechanical effects on the expression of endothelial adhesion molecules.

The vasoactive substances released by ECs are mainly the vasodilators NO and PGI_2_ and vasoconstrictors such as ET-1. The concentrations of the endothelial vasoactive substances in the medium of the carotid artery system were measured at 8, 16 and 24 h, as shown in Fig. 4c–e. The total NO metabolism was significantly increased in the circulating medium after 24 h of dynamic perfusion culturing, indicating that the physiological WSS was beneficial for increasing NO synthesis. In addition, a significant increase in PGI_2_ levels was observed, especially at 24 h. The PGI_2_ level (240.5 ± 14.67 pg/10^6^ cells) after exposure to physiological WSS at 24 h was two times higher than that at 16 h (109.9 ± 4.37 pg/10^6^ cells). However, the concentration of ET-1 at 16 h was increased relative to that at 8 h. Interestingly, the concentration of ET-1 at 24 h was decreased slightly relative to that at 16 h, indicating that the release mechanism of ET-1 is related to the duration of the WSS. WSS in the carotid artery system has been shown to stimulate NO and PGI_2_ release and inhibit ET-1 release. Prolonged physiological WSS leads to changes in the vasoactive substances released by ECs, which are ultimately responsible for regulating vascular tension [22, 23].

### The effect of carotid artery haemodynamics on endothelial permeability

**Fig. 5:**
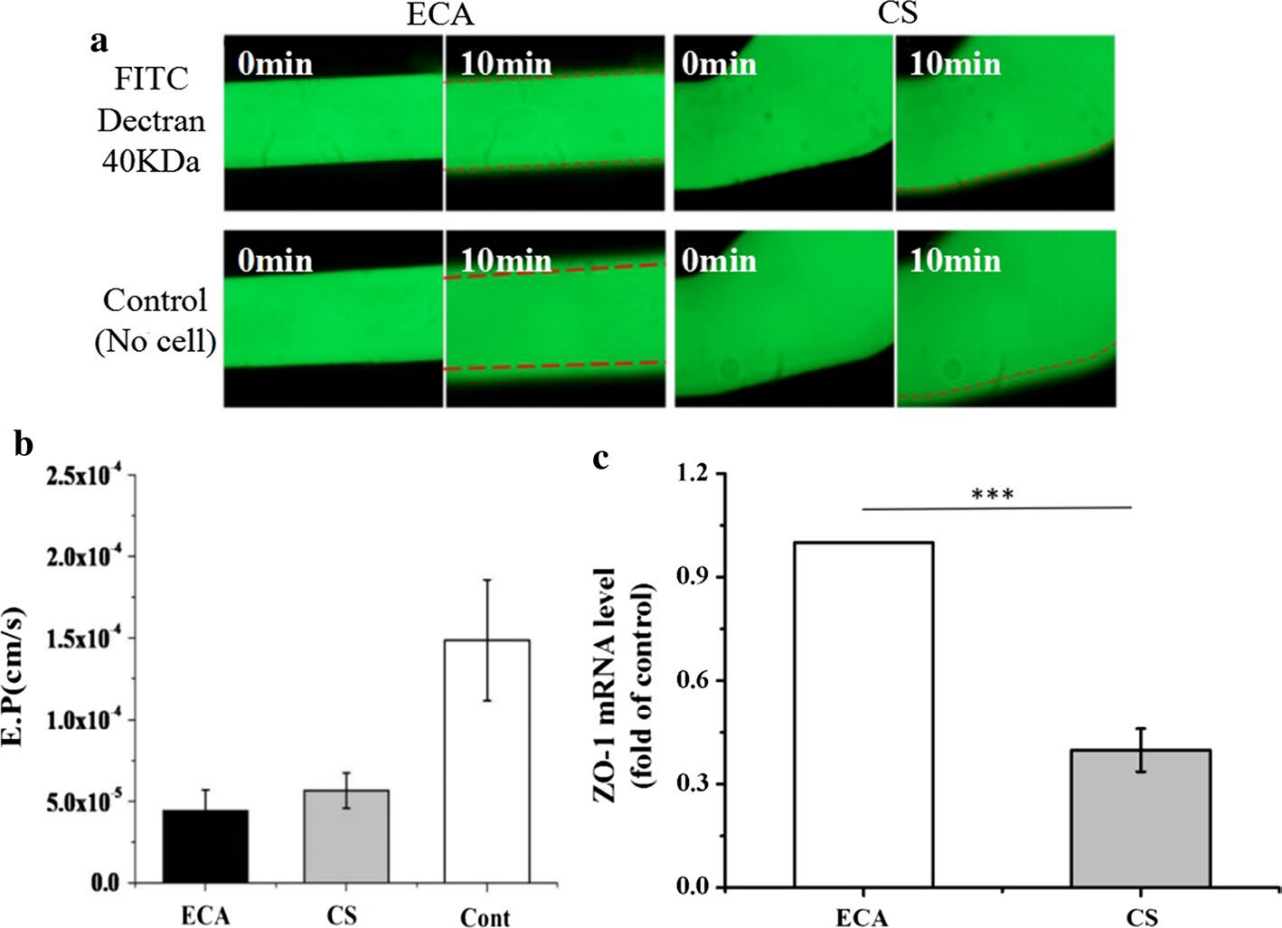
Haemodynamics effect of carotid artery on the endothelial permeability. **a** E.P images of FITC–dextran 40 kDa in the Regions ECA and CS. **b** E.P value in Regions ECA and CS with FITC–dextran 40 kDa. **c** Expression of ZO-1 was studied in the Regions ECA and CS after 24-h perfusion experiment. (Data presented are the mean ± SEM; *n* = 4; ***P < 0.001.)

Figure 5a shows the diffusion of the fluorescent tracer FITC–dextran 40 kDa in the carotid artery model after 10 min. The penetration rate was consistently higher for the control group than for the FITC–dextran 40 kDa in the different regions, which reflected the presence of the endothelial layer. The change in the fluorescence intensity was then used to calculate the E.P. As shown in Fig. 5b, the E.P values of FITC–dextran 40 kDa in Regions ECA and CS were 4.44 × 10^−5^ cm s^−1^ and 5.67 × 10^−5^ cm s^−1^, respectively. These values were lower than those in the control group (1.48 × 10^−4^ cm s^−1^). Furthermore, the ECs exposed to laminar WSS in Region ECA showed greater than 50% upregulation of ZO-1 relative to the expression in Region CS (Fig. 5c). The turbulent WSS inhibited the secretion of ZO-1, indicating its damage to ECs.

## Discussion

Haemodynamic forces, which depend on the carotid geometry, are believed to play a key role in the development of carotid artery disease. Moreover, hydrogels have been shown to maintain the biological behaviour of ECs similar to that in vivo. To the best of our knowledge, this is the first in vitro gelatin-based endothelial model that successfully replicates the TF-shaped carotid artery geometry. The gelatin-based perfusable endothelial carotid artery model provides insight into the physiological changes that occur in the endothelium of the carotid artery in vivo. The results show that the carotid artery model can be used to study variations in endothelial morphology, function and permeability that are associated with the WSS patterns of carotid artery geometry.

Natural biomaterials (collagen, gelatin, alginate fibrin and so on) are typically isolated from natural sources and have been used to improve the biological features of microvessels. Natural biomaterials provide tissue-specific biochemical and physical stimuli to guide cellular behaviours, yet they have less mechanical stability, which is why natural biomaterial hydrogels are difficult to use to construct macroscale (2–7 mm) vascular structures (e.g. carotid artery) [24]. Collagen is the main component of natural ECM and shows strong EC adhesion [25, 26]. As the denatured product of collagen, gelatin can be easily modified to improve its mechanical properties and maintain its bioactive properties. Gelatin with mechanical and biological properties will be a suitable material for constructing macroscale vascular structures. However, the gelatin exhibits weaker EC angiogenic activity because of it higher stiffness [27]. However, encouragingly, gelatin can encapsulate growth factors and achieve the controlled release of bioactive growth factors, which is beneficial to EC angiogenic activity on the gelatin [28, 29]. This is the focus of our future research.

Haemodynamics is considered an important feature for analysing atherosclerosis. Flow studies have shown that the preservation of carotid artery geometry is fundamental in reproducing in vivo WSS [15]. The risk of atherosclerotic disease is directly related to the WSS on the endothelium in the carotid artery. It has been found that low WSS rather than high WSS contributes to atherosclerosis [30, 31]. Low WSS usually occurs at complex geometries, such as bifurcating vessels or high curvature vessels. An atherogenic phenotype was found to be simulated with a carotid artery WSS below 0.4 Pa. A 3D finite element analysis based on MRI flow measurements found that the largest region of low WSS (< 0.05 Pa) occurred at the carotid sinus, while regions of high WSS (1–10 Pa) occurred at the external carotid artery [31]. As shown in Fig. 2, the difference in WSS between Regions CS and ECA was significant and representative, which facilitates the comparison of subsequent experimental results. Moreover, the low WSS of the carotid artery model occurs in Region CS.

In the laminar WSS regions, the F-actin response leads to the elongation and alignment of the F-actin cytoskeleton, as shown previously in 3D tissue culture models [32]. However, we obtained a different finding. In Region CS, the cells were significantly more rounded than those in Region ECA. The observed change to a rounded morphology in the turbulent WSS region is interesting and consistent with previous research [33, 34]. EC morphology is a key indicator of cellular health. Rounded ECs are thought to be unhealthy, whereas elongated cells are considered healthy. We infer that in Region ECA, exposure to laminar WSS is unlikely to result in the development of atherosclerotic plaques, whereas turbulent WSS regions, as in Region CS, are susceptible to the formation of atherosclerotic plaques with a cobblestone shape and disordered arrangement of ECs. Moreover, our findings indicate that in the replicated physiological environment, created by seeding the channels with a monolayer of ECs, turbulence stimulation can lead to EC aggregation in Region CS.

Under physiological conditions, ECs release a variety of molecules, including NO, PGI_2_ and ET-1. The observed enhanced NO and PGI_2_ production suggested that the mechanical forces exerted by the fluid flow affected the functional behaviour of the endothelium, consistent with observations in living vasculature. As shown in Fig. 4a, b, the increased PGI_2_ level lagged behind that of NO. NO has been shown to enhance PGI_2_ production in WSS-stimulated ECs [35]. A decrease in ET-1 production during sustained flow stimulation was observed in a population of young healthy controls [36]. The same phenomenon was observed in the flow-stimulated carotid artery model after 24 h, as shown in Fig. 4c; the ET-1 release level at 24 h was lower than that at 16 h. These results suggest that this gelatin-based perfusable endothelial carotid artery model can be used to study the regulation of the secretion of vasoactive substances under haemodynamic stimulation.

We investigated the expression of ICAM-1 and VCAM-1 in response to haemodynamics within the carotid artery model geometry. After 24 h of physiological fluid stimulation, the expression of ICAM-1 and VCAM-1 was increased significantly in Region CS relative to that in Region ECA. Carotid artery disease is influenced by the interactions of cellular adhesion molecules in response to pathological stimuli. These interactions mediate the interaction between the endothelium and blood flow and are important in the development of atherosclerosis. Endothelial dysfunction is marked by the upregulation of cellular adhesion molecules, such as ICAM-1 and VCAM-1, that cooperate with chemokines and mediate the adhesion of mononuclear and neutrophil leucocytes [37]. In pathology studies, ICAM-1 and VCAM-1 have been detected in atherosclerotic plaques and found to be upregulated in arterial ECs at lesion-prone areas in animal models. The findings of the present study indicate that ECs in Region CS are prone to dysfunction and pathological changes.

In the present study, a gelatin-based endothelial carotid artery model was used to monitor the effect of WSS on carotid artery barrier function. The WSS generated by the carotid artery geometry has both laminar and turbulent states. The pore diameter of the gelatin hydrogel is approximately 50 μm, allowing the penetration of 40 KDa FITC–dextran with a Stokes radius of 6.52 ± 0.16 nm [38], as shown in the control group in Fig. 5a. Using this model, we observed that the low WSS in Region CS increased the transendothelial E.P to FITC–dextran by almost 1.3 times than that observed in Region ECA. In addition, we monitored ZO-1 and confirmed the occurrence of WSS-induced changes in the molecules after 24 h of exposure fluid stimulus. Tight junction proteins, such as ZO-1, are thought to be the primary sites of permeability control. ZO-1 is strongly expressed in ECs that are exposed to high WSS, but less pronounced in areas with low WSS. The low WSS led to the downregulation of barrier protein expression, resulting in a sufficient gap between ECs to allow transendothelial movement of 40 KDa FITC–dextran.

The transport of molecules through the endothelium is related to the gaps in the endothelium. Some studies have shown that low WSS leads to endothelial dysfunction [39]. Moreover, WSS is required for endothelium induction and maintenance [40]. These observations implicate WSS as a putative physiological and pathological mediator of endothelium barrier function. In blood vessels, the physiologically low WSS corresponds to a shear stress with an amplitude of 0.02–1.2 Pa, which affects apoptosis and mitosis and increases the effective gap of the endothelium [41]. The physiological level of the WSS (1.5–7 Pa) appears to play a protective role in the functional integrity of ECs [42–44]. The experimental results indicated that turbulent WSS (Region CS), which increases the effective gap size, has a significant impact on the permeability of the endothelium. Moreover, the cobblestone-like ECs in Region CS displayed gap formation under WSS, and disturbed WSS caused the disorganization of endothelial integrity by gap formation [45, 46]. The findings of this study are consistent with the observation that arteriosclerotic lesions develop in areas of disturbed WSS but not laminar WSS.

## Conclusions

The study developed a gelatin-based perfusable endothelial 3D carotid artery model, which provides unique insight into the relationship between carotid artery geometry and the function of the endothelia layer. A haemodynamics simulation study showed flow velocity alterations and WSS changes due to TF-shaped carotid artery geometric variations. Hence, two representative regions (Region CS and Region ECA) in the carotid artery model were selected for analysis under laminar and turbulent WSS, respectively. Under physiological WSS for 24 h, the carotid artery model facilitated the spreading of ECs to form an endothelialized layer on the model channel surface. A progressive elongation and alignment of the ECs in the flow direction were observed in Region ECA following 8, 16 and 24 h. However, elongation of the ECs was observed in Region CS after 24 h, and the F-actin cytoskeleton remained disorganized. Further research showed that VCAM-1 and ICAM-1 expression was greatly increased in Region CS compared to that in Region ECA. Furthermore, the WSS in the carotid artery system was shown to stimulate NO and PGI_2_ release and inhibit ET-1 release after 24 h in perfusion experiments. In addition, this study showed that laminar and turbulent WSS differentially affected the morphology of the ECs. Finally, the carotid artery system in this study was proved to quantify the endothelial E.P in response to fluid mechanical forces. Low WSS induced the downregulation of ZO-1 levels compared with those under high WSS. It is envisioned that the presented carotid artery model might provide a simple way to study the relationship between WSS and endothelial dysfunction in atherosclerosis.

## Additional files

**Additional file 1.** Additional table and figures.

**Additional file 2.** Additional video.

## Data Availability

All data generated or analysed during this study are included in this published article.
