# Supplementary material for: Gelatin-based perfusable, endothelial carotid artery model for the study of atherosclerosis
Source: Biomed Eng Online. 2019 Aug 7;18:87. doi: 10.1186/s12938-019-0706-6 (PMC6685230; doi:10.1186/s12938-019-0706-6)
Supplement: Supplementary file 1 — Additional file 1. Additional table and figures. [file 12938_2019_706_MOESM1_ESM.docx]

**Additional File**

Table 1 Primers used for real-time qPCR

| **Primer name** | **Primer sequence** |
| --- | --- |
| ICAM‐1 (forward) | 5′‐TCACCTATGGCAACGACTCC‐3′ |
| ICAM‐1 (reverse) | 5′-GTGTCTCCTGGCTCTGGTTC‐3′ |
| VCAM‐1 (forward) | 5′‐GAAGGTGGCTCTGTGACCAT‐3′ |
| VCAM‐1 (reverse) | 5′‐AAAGGTGCTGTAGATTCCCATT‐3′ |
| GAPDH (forward) | 5′‐GCACCGTCAAGGCTGAGAAC‐3′ |
| GAPDH (reverse) | 5′‐TGGTGAAGACGCCAGTGGA‐3′ |


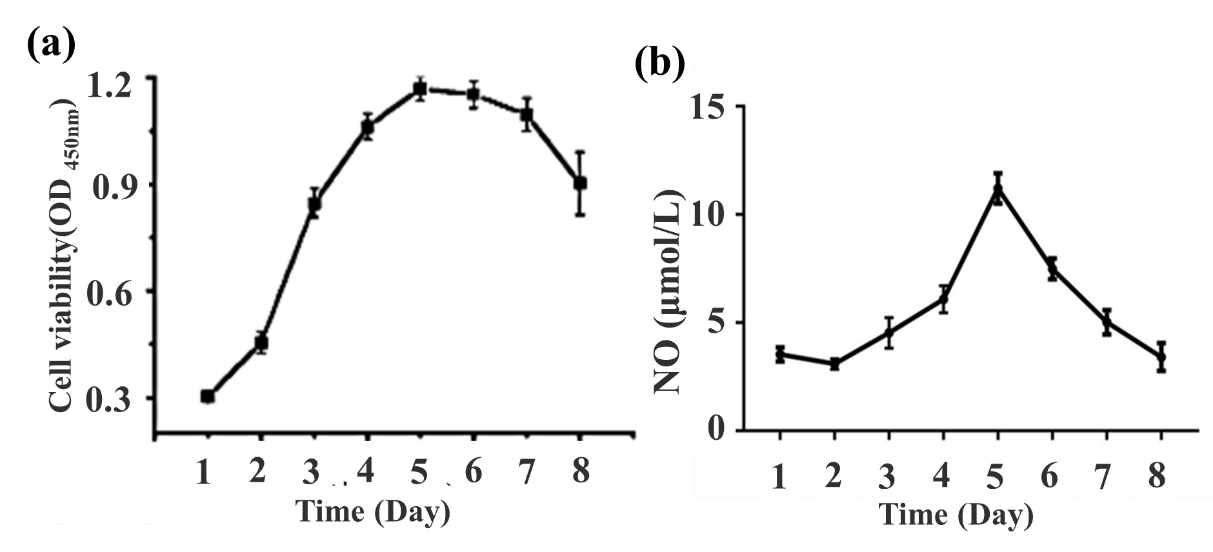


Fig.1. (a) The viability of ECs in the carotid artery model. (b) The functionality of ECs in the carotid artery model.


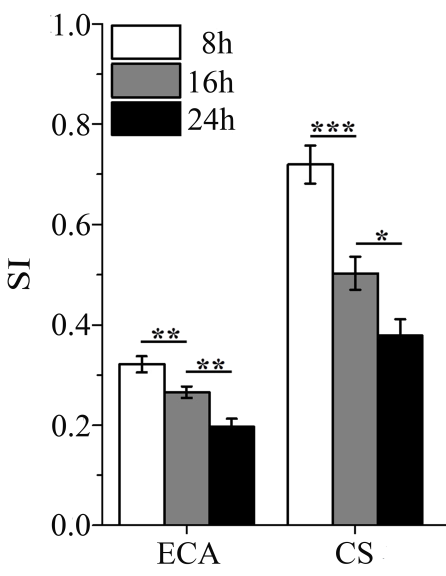


Fig.2. The SI of ECs in the carotid artery model in different study regions and time
